# Effects of Body-Oriented Interventions on Preschoolers' Social-Emotional Competence: A Systematic Review

**DOI:** 10.3389/fpsyg.2021.752930

**Published:** 2022-01-14

**Authors:** Andreia Dias Rodrigues, Ana Cruz-Ferreira, José Marmeleira, Guida Veiga

**Affiliations:** ^1^Escola de Saúde e Desenvolvimento Humano, Departamento de Desporto e Saúde, Universidade de Évora, Évora, Portugal; ^2^Comprehensive Health Research Centre, University of Évora, Évora, Portugal

**Keywords:** play, relaxation, preschool education, children, body-mind, social-emotional development

## Abstract

**Objective:**

A growing body of evidence supports the effectiveness of body-oriented interventions (BOI) in educational contexts, showing positive influences on social-emotional competence. Nevertheless, there is a lack of systematization of the evidence regarding preschool years. This is a two-part systematic review. In this first part, we aim to examine the effects of BOI on preschoolers' social-emotional competence outcomes.

**Data Sources:**

Searches were conducted in Pubmed, Scopus, PsycInfo, ERIC, Web of Science, Portal Regional da BVS and CINAHL.

**Eligibility Criteria:**

English, French and Portuguese language articles published between January 2000 and October 2020, that evaluated the effects of BOI implemented in educational contexts on social-emotional competence of preschool children. Only randomized controlled trials (RCT) or quasi-RCT were included.

**Data Extraction and Synthesis:**

Two reviewers independently completed data extraction and risk-of-bias assessment. The level of scientific evidence was measured through the Best Evidence Synthesis.

**Results:**

Nineteen studies were included. There was strong evidence that BOI do not improve anger/aggression, delay of gratification and altruism. Nevertheless, there was moderate evidence that BOI effectively improve other social-emotional outcomes, such as empathy, social interaction, social independence, general internalizing behaviors, and general externalizing behaviors. The lack of scientific evidence was compromised by the methodological quality of the studies.

**Conclusion:**

BOI effectively improve specific social-emotional competences of preschool children.

**Systematic Review Registration:**

PROSPERO, identifier CRD42020172248.

## Introduction

Early childhood is a foundational period of life for children to accomplish important milestones. In particular, during this period, there is a significant development of social-emotional competence, which is an important foundation for children's short- and long-term health, wellbeing, and success (Adela et al., 2011; Denham et al., 2012). Social-emotional competence is also crucial for children to cope with current and future stressors and challenges (Cornell et al., 2017), and contributes to academic achievement (Durlak et al., 2011).

Emotional competence refers to the adequate understanding, regulation, and expression of emotions, while social competence involves the ability of solving problems and adjusting behaviors in social situations (Denham et al., 2003). Hence, referring to social-emotional competence indicates that emotional competence and social competence work together toward adaptive behavior (Alzahrani et al., 2019).

Social-emotional competence is generally used as an umbrella term, which includes a constellation of competences that enables the expression, regulation, and comprehension of our own and others' emotions, thoughts, and behaviors in different situations, enabling the construction and maintenance of positive interpersonal relationships, and to adapt to challenge conditions (Denham et al., 2003; Denham, 2006; Alzahrani et al., 2019). The constellation of social-emotional competences has been grouped according to different models (e.g., The CASEL Model, The Big Five Model). According to the Collaborative for Academic, Social and Emotional Learning (CASEL) model, there are five core competences: self-awareness, self-management, social awareness, relationship skills, and responsible decision making (Collaborative for Academic Social and Emotional Learning, 2013). Self-awareness integrates a range of competences that allows the recognition of one's emotions, thoughts, and values that influence behaviors, and the accurate assessment of one's strengths and limitations, such as emotion expression, emotion identification, and emotion attribution (Denham, 2006; Collaborative for Academic Social and Emotional Learning, 2013; Schoon, 2021). Self-management allows the successful regulation of one's thoughts, emotions, and behaviors in various situations, and integrates competences such as self-regulation, delay of gratification, and inhibitory control (Denham, 2006; Collaborative for Academic Social and Emotional Learning, 2013; Schoon, 2021). Social awareness represents the ability of be able to understand different perspectives, empathize with others, and understand social and ethical norms for behavior, integrating competences such as empathy, peer acceptance and respect for others (Denham, 2006; Collaborative for Academic Social and Emotional Learning, 2013). Relationship skills integrate a range of competences that allows the establishment and maintenance of healthy and positive relationships, such as social competence, social interaction, and social cooperation (Denham, 2006; Collaborative for Academic Social and Emotional Learning, 2013). Lastly, responsible decision-making comprises the capacity to make constructive and positive choices about personal behavior and social interactions based on ethical and social norms, such as problem-solving skills (Denham, 2006; Collaborative for Academic Social and Emotional Learning, 2013), optimism, and purpose (Schoon, 2021).

Social-emotional competence is developed from an early age through the so-called emotion socialization process, that is, through modeling, observation, and communication about emotions with knowledgeable others (Rieffe et al., 2015). However, contexts (such as educational) that are specifically structured in the light of children's social-emotional development are also of paramount importance. Indeed, in the last few decades, several intervention programs have been implemented in educational contexts aiming to promote children's social-emotional competence (Durlak et al., 2011; Luo et al., 2020), involving different approaches, such as cognitive and behavioral (Romero-López et al., 2020; Martinsen et al., 2021), educational (Blewitt et al., 2018; Yang et al., 2019), or body-oriented (Waters et al., 2015; Gibson et al., 2017).

Body-oriented interventions (BOI) assume that bodily and emotional experiences are biologically and experientially associated. The term BOI is used in the literature, and in this review, as an umbrella term that integrates a broader range of body-oriented approaches, such as psychomotricity, play, dance, relaxation, physical activity, and exercise interventions. In general, BOI aim to provide opportunities to become aware of the body, the body in relation to others, and the connection between body and emotions (Röhricht, 2009; Probst et al., 2010; European Forum of Psychomotricity, 2012; Bellemans et al., 2017).

A growing body of evidence supports the effectiveness of BOI in the educational context, showing positive influences on social-emotional competence (Durlak et al., 2011), such as self-awareness (Chinekesh et al., 2014), self-regulation (White, 2012), social skills (Loukatari et al., 2019), as well as play behaviors (Ryalls et al., 2016).

Despite the increase of scientific evidence regarding the effects of BOI in educational contexts on children's social-emotional competence, there is a lack of systematization of the evidence regarding preschool years. The current paper is the first of a two-part systematic review. This first part aims to examine the effects of BOI on preschoolers' social-emotional competence outcomes.

## Method

The current systematic review was conducted in accordance with the Preferred Reporting Items for Systematic Review and Meta-Analysis statement (PRISMA; Moher et al., 2009) and was registered with PROSPERO, the International Prospective Register of Systematic Reviews, on July 17th, 2020 (CRD42020172248).

### Eligibility Criteria

Studies were included if they were conducted as a randomized controlled trial (RCT) or quasi-RCT, written in English, French or Portuguese, and had to be published between January 2000 and October 2020. The study participants had to attend preschool education, be between 3 and 7 years old and have typical development. The study had to focus on BOI with a minimum duration of 1 week. The study had to involve at least one comparison group, had to be delivered by humans (not computers) and implemented at school (not at home). The effects of the interventions had to be focused on participants' social-emotional competence.

### Search Strategy and Information Sources

The studies were selected for review on October 10, 2020, by searching the following databases: Pubmed, Scopus, PsycInfo, ERIC, Web of Science, Portal Regional da BVS and CINAHL. In order to identify additional potentially relevant articles, the bibliography of the selected studies was further searched by hand. The term BOI is used in the literature and in this review as an umbrella term that integrates a wider scope of therapeutic approaches (Röhricht, 2009). Search terms related to BOI (i.e., “body-oriented,” “psychomotor,” “dance movement,” “psychomotor physiotherapy,” “mind-body,” “movement oriented,” “body awareness,” and “body psychotherapy”) as well as terms associated with a broader collection of physiological interventions regularly used by psychomotor therapists (Probst et al., 2010; European Forum of Psychomotricity, 2012; Bellemans et al., 2017) (i.e., “exercise,” “physical training,” “sport,” “running,” “physiotherapy,” “yoga,” “relaxation,” and “play”) were paired with terms related to social-emotional competence (i.e., “socio-emotional,” “socioemotional,” “social-emotional,” “social,” “emotion,” “soft,” and “non-cognitive”) and terms concerning preschool age (i.e., “school,” “preschool,” “kindergarten,” “nursery,” “pre-K,” and “playgroup”).

### Study Selection

The study selection process followed PRISMA guidelines. First, two reviewers (ADR and GV) independently read all abstracts and classified them as excluded or potentially included. A third reviewer (JM) was consulted if there was disagreement between the two reviewers. To increase the chances of finding important information, the search team also checked through the included studies' reference lists to verify whether these references included other studies that could be eligible for the review. Reviewers applied the inclusion criteria after reading the full texts of the potentially included studies. The research team contacted the corresponding author if there was any lack of clarity regarding the information provided in any article, or if there was a lack of information.

### Data Extraction

Two researchers (ADR and GV) extracted the data from the selected papers. The extracted data included authors, year of publication, study type and design, subjects, intervention used, outcomes measures and key outcomes results. The third reviewer (JM) was consulted to resolve disagreements between the two reviewers.

### Risk of Bias Assessment

The methodological quality of the studies was assessed independently by two reviewers (ADR and ACF), using the Physiotherapy Evidence Database (PEDro) scale (de Morton, 2009), with the third (JM) reviewer consulted to resolve disagreements (11%). The PEDro scale is based on the Delphi list developed by Verhagen et al. (1998). It consists of 11 items, including specified eligibility criteria, random allocation, concealed allocation, baseline comparability, blinding of subjects, blinding of therapists, blinding of assessors, adequate follow-up, intention-to-treat analysis, between-group statistical comparisons, and point estimates and variability. The eligibility criterion is related to external validity and is not used to calculate the PEDro score. For each study included, a PEDro sum score ranging from 1 to 10 could be obtained, with higher scores indicating better methodological quality. To the best of our knowledge, there are no published validated cutoff scores for the PEDro scale. Therefore, the following criteria were used to rate method quality: a score of <5 indicates “low quality”, and a score of 5 or higher indicates “high quality” (Silva et al., 2012; Martins et al., 2016; Pastora-Bernal et al., 2017).

### Data Synthesis

The level of the scientific evidence was measured through the Best Evidence Synthesis (BES) (Slavin, 1986) by two researchers (ADR and ACF). BES is an alternative to meta-analysis and seeks to apply consistent, well-justified standards to identify unbiased, meaningful information from experimental studies (Slavin, 1986). The following criteria were used to grade the strength of the evidence: strong evidence, obtained in multiple high-quality RCTs; moderate evidence, obtained in one high-quality RCT and one or more low-quality RCTs; limited evidence, obtained in one high-quality or multiple low-quality RCTs; and no evidence, trough one low-quality RCT or contradictory outcomes (Van Tulder et al., 1997).

## Results

### Study Selection

**Figure 1:**
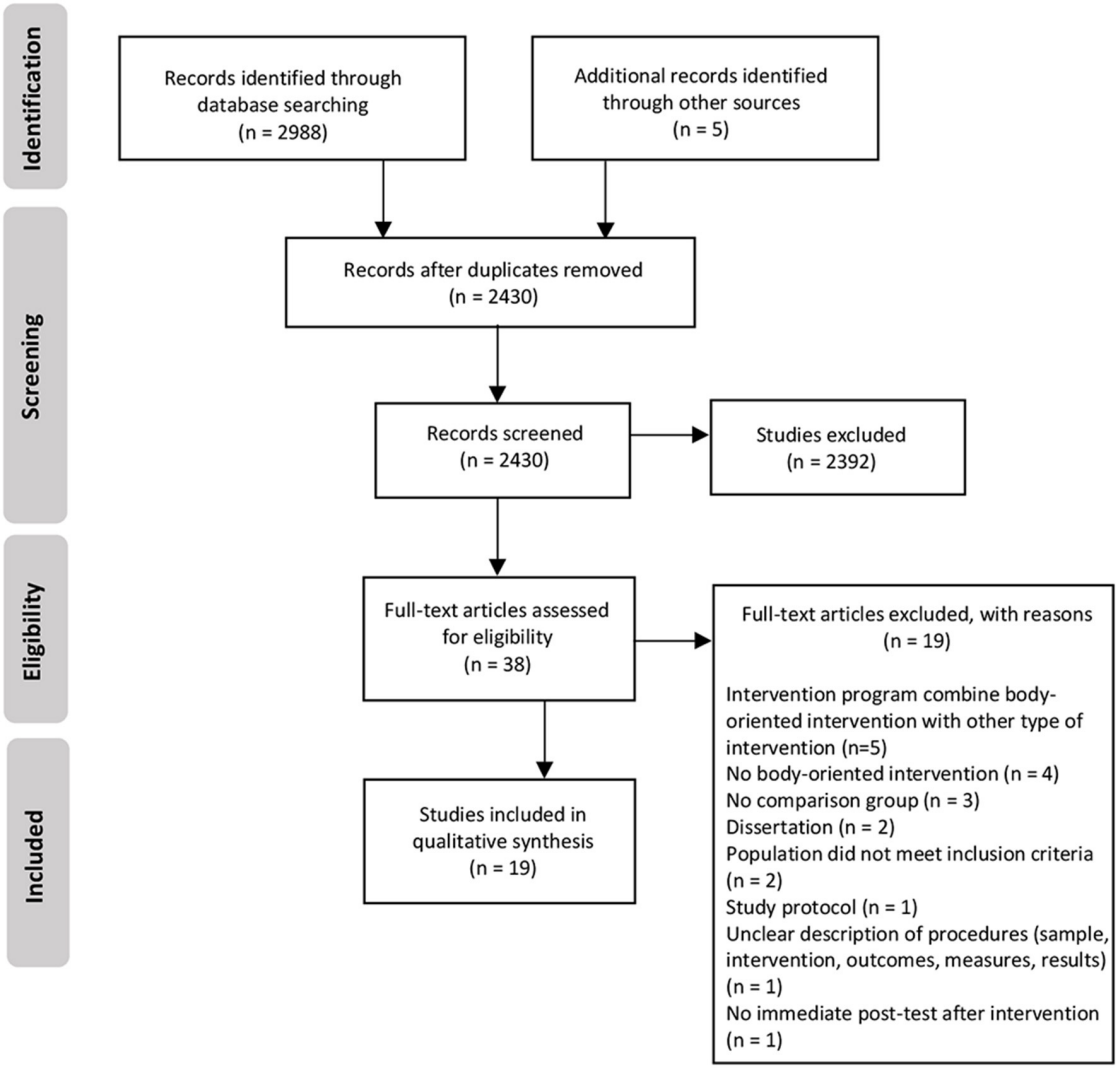
Flow diagram.

We summarized the flow of literature search and study selection in Figure 1. Electronic and reference searches generated 2,991 records, including 563 duplicate articles that were removed. The remaining 2,428 articles were further screened (title and abstract), and among these, 2392 were excluded based on not meeting our study criteria. When 38 full-text articles were further assessed for eligibility, we read each article, and found 19 articles that did not meet the predetermined inclusion criteria: the intervention program combines body-oriented intervention with non-body oriented intervention (*n* = 5); no body-oriented intervention (*n* = 4); no comparison group (*n* = 3); dissertation (*n* = 2); population does not meet the inclusion criteria (*n* = 2); study protocol (*n* = 1); unclear description of procedures (sample, intervention, outcomes, measures, results) (*n* = 1), and no immediate post-test after intervention (*n* = 1). In total, 19 studies were considered for the qualitative synthesis.

### Study Characteristics

We summarized all the eligible studies of this systematic review in Table 1. These 19 selected studies (English = 17 and French = 2) were published between 2006 and 2020. Study types included RCTs (*n* = 14) and quasi-RCT (*n* = 5). The most frequent study design was the pre-post test (*n* = 17). One study included follow-up (Cheng and Ray, 2016), and another used an additional measurement during the study intervention (Solomon et al., 2018). Study sample sizes ranged from 19 to 372 participants. Participants' ages ranged from 3 to 7 years. All the studies included 2 groups (experimental and control groups), except in the studies by Goldstein and Lerner (2017), Murray et al. (2018), in which 3 groups were used (1 experimental group and 2 active control groups, and 1 experimental group, 1 active and 1 inactive control groups, respectively). All the studies used BOI as the study intervention. Control groups were inactive in 17 studies (Lobo and Winsler, 2006; Ortega et al., 2009; Hashemi et al., 2012; Chinekesh et al., 2014; Flook et al., 2015; Ozyurek et al., 2015; Anna et al., 2016; Biber, 2016; Cheng and Ray, 2016; Robinson et al., 2016; Murray et al., 2018; Duman and Ozkur, 2019; Loukatari et al., 2019; Richard et al., 2019; Deneault et al., 2020; Lee et al., 2020; Tersi and Matsouka, 2020). In the remaining 2 studies, the BOI was compared with block building and story time reading activities (Goldstein and Lerner, 2017), and a play-based program following a teacher-directed approach (Solomon et al., 2018).

**Table 1:** Characteristics of the included studies in the review.

**Study**	**Study type/design**	**Subjects**	**Intervention**	**Measures/Outcomes**	**Results**
Lobo and Winsler (2006)	RCT Pre-post test	*N* = 40; age range, 3–5 Body-oriented group: *n* = 21 Control group: *n* = 19	Duration and frequency: 8 week, 2 × 35′ per week Body-oriented group = creative dance/movement program Control group = no intervention	Social Competence Behavior Evaluation—Preschool Edition^c, e^ = social competence; general internalizing behaviors; general externalizing behaviors	Body-oriented group = improved social competence, general internalizing behaviors and general externalizing behaviors Control group = no differences
Ortega et al. (2009)	RCT Pre-post test	*N* = 45; age range, 5–6 Body-oriented group: *n* = 22 Control group: *n* = 23	Duration and frequency: 13 week, 1 × 300′ per week Body-oriented group = group play activities program Control group = no intervention	Sociogrammes^b^ = play Mappings—Child-directed play^d^ = number of groups; group size; group composition; interaction with communication (forms of interaction) Mappings—Teacher-directed play^d^ = number of groups; group size; group composition; interaction with communication (forms of interaction)	Body-oriented group = improved play. On child-directed play, improved interaction with communication; no differences on number of groups, group size and group composition. On teacher-directed play, improved number of groups and interaction with communication; no differences on group size and group composition Control group = no differences on play. On child-directed play, increased solitary play without communication (forms of interaction); no differences on number of groups, group size, and group composition. On teacher-directed play, no differences on number of groups, group size, group composition and interaction with communication
Hashemi et al. (2012)	RCT Pre-post test	*N* = 60; age range, 3–6 Body-oriented group: *n* = 30 Control group: *n* = 30	Duration and frequency: 12 week, 2 × 60′ per week Body-oriented group = gymnastics program Control group = no intervention	Preschool and Kindergarten Behavior Scale^c^ = social cooperation; social interaction; social independence; social competence	Body-oriented group = improved social cooperation, social interaction, social independence, and social competence Control group = no differences
Chinekesh et al. (2014)	RCT Pre-post test	*N* = 372; mean age, 5.1 Body-oriented group: *n* = 186 Control group: *n* = 186	Duration and frequency: 5 week, 3 × 90′ per week Body-oriented group = group play therapy program Control group = no intervention	Social-emotional Questionnaire^c^ = self-awareness; self-regulation; social competence; empathy; social-emotional competence	Body-oriented group = improved self-awareness, self-regulation, social competence, empathy, and social-emotional competenceControl group = no differences
Flook et al. (2015)	RCT Pre-post test	*N* = 68; mean age, 4.67 Body-oriented group: *n* = 30 Control group: *n* = 38	Duration and frequency: 12 week, 2 × 20–30′ per week Body-oriented group = mindfulness-based activities program Control group = no intervention	Teacher Social Competence Scale^e^ = prosocial behavior; emotion regulation; social competence School grades records^e^ = social-emotional competence Sharing task^a^ = sharing Delay of gratification task^a^ = delay of gratification Dimensional Change Card Sort Task^a^ = cognitive flexibility Flanker Task^a^ = inhibitory control	Body-oriented group = improved prosocial behavior, emotion regulation, social competence, social-emotional competence and sharing; no differences on delay of gratification, cognitive flexibility, and inhibitory control Control group = improved prosocial behavior, emotion regulation, social competence, and decreased sharing; no differences on delay of gratification, cognitive flexibility, inhibitory control, and social-emotional competence
Ozyurek et al. (2015)	RCT Pre-post test	*N* = 42; average age, 4 Body-oriented group: *n* = 21 Control group: *n* = 21	Duration and frequency: 14 week, 2 × 30–50′ per week Body-oriented group = game-based activities program Control group = no intervention	Preschool Social Skills Rating Scale^c, e^ = friendship skills; emotion regulation	Body-oriented group = improved friendship skills and emotion regulation Control group = improved friendship skills and emotion regulation
Anna et al. (2016)	RCT Pre-post test	*N* = 29; age range, 3–5 Body-oriented group: *n* = 14 Control group: *n* = 15	Duration and frequency: 8 week, 2 × 40′ per week Body-oriented group = psychomotor program Control group = no intervention	Pictorial Scale of Perceived Competence and Social Acceptance for Young Children^b^ = peer acceptance	Body-oriented group = no differences Control group = no differences
Biber (2016)	Quasi-RCT Pre-post test	*N* = 40; age range, 5–6 Body-oriented group: *n* = 20 Control group: *n* = 20	Duration and frequency: 8 week, 4 × 40′ per week Body-oriented group = folk dance program Control group = no intervention	Social Adjustment and Skills Scale^e^ = social competence	Body-oriented group = improved social competence Control group = no differences
Cheng and Ray (2016)	RCT Pre-post test 1-month follow-up	*N* = 43; age range 5–6 Body-oriented group: *n* = 21 Control group: *n* = 22	Duration and frequency: 8 week, 2 × 30′ per week Body-oriented group = child-centered group play therapy program Control group = no intervention	Social Emotional Assets and Resilience Scale—Parent^c^ = self-regulation; social competence; empathy; social-emotional competence Social Emotional Assets and Resilience Scale—Teacher^e^ = social-emotional competence	Body-oriented group = improved social competence, empathy, and social-emotional competence (reported by parents); no differences on self-regulation and social-emotional competence (reported by teachers). Control group = no differences
Robinson et al. (2016)	RCT Pre-post test	*N* = 113; mean age, 4.01 Body-oriented group: *n* = 68 Control group: *n* = 45	Duration and frequency: 5 week, 3 × 40′ per week Body-oriented group = motor skills intervention program Control group = no intervention	Delay of Gratification Snack Task - Preschool Self-Regulation Assessment^a^ = delay of gratification	Body-oriented group = no differences Control group = decreased delay of gratification
Goldstein and Lerner (2017)	RCT Pre-post test	*N* = 86; age range, 4–5 Body-oriented group: *n* = 31 Control group 1: *n* = 29 Control group 2: *n* = 26	Duration and frequency: 8 week, 3 × 30′ per week Body-oriented group = pretend play games program Control group 1 = block building activities program Control group 2 = story time reading intervention program	Theory of Mind Scale^a^ = Theory of Mind Sticker “Dictator Game” ^a^ = altruism Berkeley Puppet Interview Method^a^ = emotion attribution Live hurt protocols—Adapted^d^ = emotion regulation; prosocial behavior Social Interaction Observation System^d^ = social interaction	Body-oriented group = decreased emotion attribution; improved emotion regulation, social interaction; no differences on Theory of Mind, altruism, and prosocial behavior Control group 1 = no differences Control group 2 = no differences
Murray et al. (2018)	RCT Pre-post test	*N* = 101; mean age, 6.24 Body-oriented group: *n* = 33 Attention training group: *n* = 30 Control group: *n* = 38	Duration and frequency: 1 week, 3 × 11′ per week Body-oriented group = progressive muscle relaxation program Attention training group = attention training technique program Control group = no intervention.	Marshmallow Test^a^ = delay of gratification Day/Night Task^a^ = inhibitory control	Body-oriented group = no differences Attention training group = improved delay of gratification, and inhibitory control Control group = no differences
Solomon et al. (2018)	RCT Pre- mid- and post test (0, 8 m, 15 m)	*N* = 256; age range, 3–4 Body-oriented group = 148 Control group = 108	Duration and frequency: 60 week, 5 × integrated on preschool curriculum Body-oriented group = play-based program using a teacher-directed approach Control group = play-based program using a child-centered approach	Day/Night Task^a^ = inhibitory control Head-To-Toes Task^a^ = inhibitory control Strengths and Difficulties Questionnaire^c, e^ = total of difficulties Social Competence Behavior Evaluation—Preschool Edition^e^ = social competence; anger/aggression; anxiety/withdrawal	Body-oriented group = children with high levels of initial hyperactivity/inattention, improved on inhibitory control (Head-To-Toes Task); no differences on inhibitory control (Day/Night Task), social competence, anger/aggression and anxiety/withdrawal Control group = no differences
Duman and Ozkur (2019)	Quasi-RCT Pre-post test	*N* = 30; average age, 5 Body-oriented group: *n* = 15 Control group: *n* = 15	Duration and frequency: 12 week, 5 × 45-60′ per week Body-oriented group = embedded learning-based movement education program Control group = no intervention	Child Behavior Rating Scale^d^ = self-regulation	Body-oriented group = improved self-regulation (greater than control group). Control group = improved self-regulation
Loukatari et al. (2019)	RCT Pre-post test	*N* = 60; age range, 5–6 Body-oriented group: *n* = 30 Control group: *n* = 30	Duration and frequency: 4 week, 3 × 30′ per week Body-oriented group = structured playful activities program Control group = no intervention	Social Skills Rating System^e^ = social cooperation; social assertion; self-regulation; general externalizing behaviors; general internalizing behaviors; hyperactivity; social competence	Body-oriented group = improved social cooperation, social assertion, self-regulation, general externalizing behaviors, general internalizing behaviors, hyperactivity, and social competence Control group = no differences
Richard et al. (2019)	RCT Pre-post test	*N* = 19; mean age, 5.7 Body-oriented group: *n* = 9 Control group: *n* = 10	Duration and frequency: 11 week, 1 × 60′ per week Body-oriented group = pretend play-based program Control group = no intervention	Emotional Vocabulary Test^a^ = emotion recognition Perceptual Identification of Emotional Facial Expressions Task^a^ = emotion identification; emotion attribution (anger, disgust, fear, and sadness identification) Comprehension of Causes of Emotions Task^a^ = emotion attribution Contextual Task^a^ = emotion attribution Structured Interview about strategies for regulating negative emotions^b^ = functional emotion regulation strategies; dysfunctional emotion regulation strategies Emotion Regulation Checklist^c^ = emotion regulation Altruistic Initiatives Task^a^ = altruism Challenging Situation Task—Revised^a^ = prosocial behavior; anger/aggression; social avoidance	Body-oriented group = improved emotion recognition, emotion attribution and emotion attribution on anger, disgust, fear, and sadness identification, and dysfunctional emotion regulation strategies; no differences on emotion identification, emotion attribution (Comprehension of Causes of Emotions Task), functional emotion regulation strategies, emotion regulation, altruism, prosocial behavior, anger/aggression, and social avoidance Control group = improved emotion attribution (Perceptual Identification of Emotional Facial Expressions Task), and altruism; no differences on emotion recognition, emotion attribution (Comprehension of Causes of Emotions Task and Contextual Task), emotion identification, functional and dysfunctional emotion regulation strategies, emotion regulation, prosocial behavior, anger/aggression, and social avoidance
Deneault et al. (2020)	Quasi-RCT Pre-post test	*N* = 76; age range, 4–7 Body-oriented group: *n* = 45 Control group: *n* = 31	Duration and frequency: 6 week, 1 × 60′ per week Body-oriented group = symbolic sand play program Control group = no intervention	Strengths and Difficulties Questionnaire^e^ = general internalizing behaviors; behavior problems; hyperactivity; peer relations problems; prosocial behavior	Body-oriented group = improved general internalizing behaviors, behavior problems, hyperactivity, peer relations problems, and prosocial behavior Control group = improved hyperactivity; no differences on general internalizing behaviors, behavior problems, peer relations problems, and prosocial behavior
Lee et al. (2020)	Quasi-RCT Pre-post test	*N* = 42; age range, 4–6 Body-oriented group: *n* = 20 Control group: *n* = 22	Duration and frequency: 1 week, 5 × 70′ per week Body-oriented group = loose parts play and mindfulness activities program Control group = no intervention	Smiley Face Likert Scale^b^ = happiness after play Children's Emotional Manifestation Scale^d^ = emotion expression Penn Interactive Peer Play Scale^d^ = play disruption; play disconnection; play interaction Test of Playfulness Scale^d^ = play extent; play intensity; play skill	Body-oriented group = improved happiness after play, play disruption, play disconnection, play interaction, play extent, play intensity and play skill; no differences on emotion expression Control group = improved play extent, play intensity and play skill; no differences on happiness after play, emotion expression, play disruption, play disconnection, and play interaction
Tersi and Matsouka (2020)	Quasi-RCT Pre-post test	*N* = 40; age range, 4–6 Body-oriented group: *n* = 20 Control group: *n* = 20	Duration and frequency: 4 week, 2 × 45′ per week Body-oriented group = structured play activities program Control group = no intervention	Preschool Kindergarten Behavior Scale^e^ = social cooperation; social independence; social interaction; social competence; general externalizing behaviors; general internalizing behaviors; behavior problems	Body-oriented group = improved social cooperation, social independence, social interaction, social competence, general externalizing behaviors, general internalizing behaviors, and behavior problems Control group = improved social cooperation, social interaction, social competence, general internalizing behaviors, and behavior problems; no differences on social independence and general externalizing behaviors

a *Applied to children*;

b *Reported by children*;

c *Reported by parents*;

d *Reported by researchers*;

e *Reported by teachers*.

### BOI Characteristics

The BOI duration and frequency ranged from 5 days to 60 weeks and from a 1 to a 5 times per week basis. The duration of the BOI sessions ranged from 11 to 300 mins. As we mentioned, BOI is an umbrella term that integrates a wider range of therapeutic approaches (Röhricht, 2009). The types of BOI in the included studies were creative dance/movement (Lobo and Winsler, 2006), group play activities (Ortega et al., 2009), gymnastics (Hashemi et al., 2012), group play therapy (Chinekesh et al., 2014), mindfulness-based activities (Flook et al., 2015), game-based activities (Ozyurek et al., 2015), psychomotor program (Anna et al., 2016), folk dance (Biber, 2016), child-centered group play (Cheng and Ray, 2016), motor skills intervention (Robinson et al., 2016), pretend play games (Goldstein and Lerner, 2017), progressive muscle relaxation (Murray et al., 2018), play-based activities using a teacher-centered approach (Solomon et al., 2018), embedded learning-based movement education (Duman and Ozkur, 2019), structured playful activities (Loukatari et al., 2019), pretend play-based (Richard et al., 2019), symbolic sand play (Deneault et al., 2020), loose parts play and mindfulness activities (Lee et al., 2020), and structured play activities (Tersi and Matsouka, 2020).

### Effects of BOI on Social-Emotional Competence Outcomes

Outcomes measures are noted in Table 1 in the “Measures/Outcomes” column. Following Mayo-Wilson et al. (2017), Saldanha et al. (2020) and colleagues' suggestions, the organization of the outcomes (see Table 2) took into consideration the respective measure and definition. For a better systematization of the findings, the outcomes were grouped into the following categories: social-emotional outcomes; child's play; and child's behaviors.

**Table 2:** Summary of the outcomes of the studies.

**Final outcome**		**Study outcome**	**Measures**	**Study**
Social-emotional competence		Social-emotional skills	Social-emotional Questionnaire (Miller et al., 2005)	Chinekesh et al. (2014)
		Social and emotional development	School Grades Records (Flook et al., 2015)	Flook et al. (2015)
		Social-emotional assets total score	Social Emotional Assets and Resilience Scales (Merrell, 2011)	Cheng and Ray (2016)
**CASEL categories**				
Self-awareness	Self-awareness	Self-awareness	Social-emotional Questionnaire (Miller et al., 2005)	Chinekesh et al. (2014)
	Emotion expression	Emotional behaviors	Children's Emotional Manifestation Scale (Li and Lopez, 2005)	Lee et al. (2020)
Self-management	Emotion regulation	Emotion regulation	Teacher Social Competence Scale (Conduct Problems Prevention Research Group, 1995)	Flook et al. (2015)
		Emotional management skills	Preschool Social Skills Rating Scale (Omeroglu et al., 2015)	Ozyurek et al. (2015)
		Live distress response	Live Hurt Protocols—Adapted (Goldstein and Lerner, 2017)	Goldstein and Lerner (2017)
		Emotion regulation	Emotion Regulation Checklist (Nader-Grosbois and Mazzone, 2015)	Richard et al. (2019)
	Functional emotion regulation strategies	Functional emotion regulation strategies	Structured Interview about strategies for regulating negative emotions (López-Pérez et al., 2017)	Richard et al. (2019)
	Dysfunctional emotion regulation strategies	Dysfunctional emotion regulation strategies	Structured Interview about strategies for regulating negative emotions (López-Pérez et al., 2017)	Richard et al. (2019)
	Self-regulation	Self-regulation	Social-emotional Questionnaire (Miller et al., 2005)	Chinekesh et al. (2014)
		Self-regulation	Social Emotional Assets and Resilience Scales (Merrell, 2011)	Cheng and Ray (2016)
		Self-regulation	Child Behavior Rating Scale (Sezgin, 2016)	Duman and Ozkur (2019)
		Self-control	Social Skills Rating System (Gresham and Elliott, 1990)	Loukatari et al. (2019)
	Delay of gratification	Delay of gratification	Delay of Gratification Task (Prencipe and Zelazo, 2005)	Flook et al. (2015)
		Delay of gratification	Delay of Gratification Snack Task - Preschool Self- Regulation Assessment (Smith-Donald et al., 2007)	Robinson et al. (2016)
		Delay of gratification	Marshmallow Test (Mischel and Ebbesen, 1970)	Murray et al. (2018)
	Inhibitory control	Inhibitory control	Flanker Task (Zelazo et al., 2013)	Flook et al. (2015)
		Verbal inhibition	Day/Night task (Gerstadt et al., 1994)	Murray et al. (2018)
		Inhibitory control	Head-To-Toes Task (Ponitz et al., 2009)	Solomon et al. (2018)
		Verbal inhibition	Day/Night Task (Gerstadt et al., 1994)	Solomon et al. (2018)
	Cognitive flexibility	Cognitive flexibility	Dimensional Change Card Sort Task (Garon et al., 2008)	Flook et al. (2015)
	General internalizing behaviors	Internalizing behavior problems	Social Competence Behavior Evaluation - Preschool Edition (LaFreniere and Dumas, 1995)	Lobo and Winsler (2006)
		Internalizing	Social Skills Rating System (Gresham and Elliott, 1990)	Loukatari et al. (2019)
		Emotional symptoms	Strengths and Difficulties Questionnaire (Goodman, 2001)	Deneault et al. (2020)
		Internalizing problems	Preschool and Kindergarten Behavior Scale (Merrell, 2002)	Tersi and Matsouka (2020)
	Anxiety/withdrawal	Anxiety/withdrawal	Social Competence Behavior Evaluation - Preschool Edition (LaFreniere and Dumas, 1995)	Solomon et al. (2018)
	General externalizing behaviors	Externalizing behavior problems	Social Competence Behavior Evaluation - Preschool Edition (LaFreniere and Dumas, 1995)	Lobo and Winsler (2006)
		Externalizing	Social Skills Rating System (Gresham and Elliott, 1990)	Loukatari et al. (2019)
		Externalizing problems	Preschool and Kindergarten Behavior Scale (Merrell, 2002)	Tersi and Matsouka (2020)
	Anger/aggression	Anger/aggression	Social Competence Behavior Evaluation - Preschool Edition (LaFreniere and Dumas, 1995)	Solomon et al. (2018)
		Aggression	Challenging Situation Task—Revised (Denham et al., 1994)	Richard et al. (2019)
	Behavior problems	Conduct problems	Strengths and Difficulties Questionnaire (Goodman, 2001)	Deneault et al. (2020)
		Behavior problems total score	Preschool and Kindergarten Behavior Scale (Merrell, 2002)	Tersi and Matsouka (2020)
	Hyperactivity	Hyperactivity	Social Skills Rating System (Gresham and Elliott, 1990)	Loukatari et al. (2019)
		Hyperactivity	Strengths and Difficulties Questionnaire (Goodman, 2001)	Deneault et al. (2020)
Social awareness	Empathy	Empathy	Social-emotional Questionnaire (Miller et al., 2005)	Chinekesh et al. (2014)
		Empathy	Social Emotional Assets and Resilience Scales (Merrell, 2011)	Cheng and Ray (2016)
	Theory of Mind	Theory of Mind	Theory of Mind Scale (Wellman and Liu, 2004)	Goldstein and Lerner (2017)
	Emotion identification	Emotion expression identification	Perceptual Identification of Emotional Facial Expressions Task (Theurel et al., 2016)	Richard et al. (2019)
	Emotion recognition	Emotion recognition	Emotional Vocabulary Task (Theurel and Gentaz, 2015)	Richard et al. (2019)
	Emotion attribution	Emotion matching	Berkeley Puppet Interview Method (Bryant, 1982)	Goldstein and Lerner (2017)
		Emotion attribution (anger, disgust, fear, and sadness identification)	Perceptual Identification of Emotional Facial Expressions Task (Theurel et al., 2016)	Richard et al. (2019)
		Emotion attribution	Contextual Task (Korkman et al., 2012)	Richard et al. (2019)
		Comprehension of causes of emotion	Comprehension of Causes of Emotions Task (Theurel and Gentaz, 2015)	Richard et al. (2019)
Relationship skills	Social competence	Social competence	Social Competence Behavior Evaluation - Preschool Edition (LaFreniere and Dumas, 1995)	Lobo and Winsler (2006)
		Social skills total score	Preschool and Kindergarten Behavior Scale (Merrell, 2002)	Hashemi et al. (2012)
		Adaptive behavior	Social-emotional Questionnaire (Miller et al., 2005)	Chinekesh et al. (2014)
		Social adjustment	Social-emotional Questionnaire (Miller et al., 2005)	Chinekesh et al. (2014)
		Social competence total score	Teacher Social Competence Scale (Conduct Problems Prevention Research Group, 1995)	Flook et al. (2015)
		Social adjustment total score	Social Adjustment and Skills Scale (Omeroglu and Kandir, 2005)	Biber (2016)
		Social competence	Social Emotional Assets and Resilience Scales (Merrell, 2011)	Cheng and Ray (2016)
		Social competence	Social Competence Behavior Evaluation—Preschool Edition (LaFreniere and Dumas, 1995)	Solomon et al. (2018)
		Social skills total score	Social Skills Rating System (Gresham and Elliott, 1990)	Loukatari et al. (2019)
		Social skills total score	Preschool and Kindergarten Behavior Scale (Merrell, 2002)	Tersi and Matsouka (2020)
	Social interaction	Social interaction	Preschool and Kindergarten Behavior Scale (Merrell, 2002)	Hashemi et al. (2012)
		Classroom social behavior	Social Interaction Observation System (Bauminger, 2002)	Goldstein and Lerner (2017)
		Social interaction	Preschool and Kindergarten Behavior Scale (Merrell, 2002)	Tersi and Matsouka (2020)
	Social cooperation	Social cooperation	Preschool and Kindergarten Behavior Scale (Merrell, 2002)	Hashemi et al. (2012)
		Cooperation	Social Skills Rating System (Gresham and Elliott, 1990)	Loukatari et al. (2019)
		Social cooperation	Preschool and Kindergarten Behavior Scale (Merrell, 2002)	Tersi and Matsouka (2020)
	Social independence	Social independence	Preschool and Kindergarten Behavior Scale (Merrell, 2002)	Hashemi et al. (2012)
		Social independence	Preschool and Kindergarten Behavior Scale (Merrell, 2002)	Tersi and Matsouka (2020)
	Social assertion	Assertion	Social Skills Rating System (Gresham and Elliott, 1990)	Loukatari et al. (2019)
	Friendship skills	Friendship skills	Preschool Social Skills Rating Scale (Omeroglu et al., 2015)	Ozyurek et al. (2015)
	Altruism	Altruism	Sticker “Dictator Game” (Blake and Rand, 2010)	Goldstein and Lerner (2017)
		Altruism	Altruistic Initiatives Task (Bryan et al., 2014)	Richard et al. (2019)
	Sharing	Sharing	Sharing Task (Flook et al., 2015)	Flook et al. (2015)
	Social avoidance	Avoidance behavior	Challenging Situation Task—Revised (Denham et al., 1994)	Richard et al. (2019)
	Peer relations problems	Peer relations problems	Strengths and Difficulties Questionnaire (Goodman, 2001)	Deneault et al. (2020)
	Peer acceptance	Peer acceptance	Pictorial Scale of Perceived Competence and Social Acceptance for Young Children (Makri-Botsari, 2001)	Anna et al. (2016)
	Prosocial behavior	Prosocial behavior	Teacher Social Competence Scale (Conduct Problems Prevention Research Group, 1995)	Flook et al. (2015)
		Helping behaviors	Live Hurt Protocols—Adapted (Goldstein and Lerner, 2017)	Goldstein and Lerner (2017)
		Comforting behaviors	Live Hurt Protocols—Adapted (Goldstein and Lerner, 2017)	Goldstein and Lerner (2017)
		Prosocial behavior	Challenging Situation Task—Revised (Denham et al., 1994)	Richard et al. (2019)
		Prosocial behavior	Strengths and Difficulties Questionnaire (Goodman, 2001)	Deneault et al. (2020)
**Play behavior**				
	Play	Play	Sociogrammes (Ortega et al., 2009)	Ortega et al. (2009)
	Interaction with communication on child-directed play	Forms of interaction—child-directed play	Mappings (Ortega et al., 2009)	Ortega et al. (2009)
	Number of groups on teacher-directed play	Number of groups—teacher-directed play	Mappings (Ortega et al., 2009)	Ortega et al. (2009)
	Interaction with communication on teacher-directed play	Forms of interaction– teacher-directed play	Mappings (Ortega et al., 2009)	Ortega et al. (2009)
	Play disruption	Play disruption	Penn Interactive Peer Play Scale (Fantuzzo et al., 1998)	Lee et al. (2020)
	Play disconnection	Play disconnection	Penn Interactive Peer Play Scale (Fantuzzo et al., 1998)	Lee et al. (2020)
	Play interaction	Play interaction	Penn Interactive Peer Play Scale (Fantuzzo et al., 1998)	Lee et al. (2020)
	Happiness after play	Happiness after play	Smiley Face Likert Scale (Hall et al., 2016)	Lee et al. (2020)
	Play extent	Play extent	Test of Playfulness Scale (Bundy, 1997)	Lee et al. (2020)
	Play intensity	Play intensity	Test of Playfulness Scale (Bundy, 1997)	Lee et al. (2020)
	Play skill	Play skill	Test of Playfulness Scale (Bundy, 1997)	Lee et al. (2020)
	Group composition on child-directed play	Group composition—child-directed play	Mappings (Ortega et al., 2009)	Ortega et al. (2009)
	Number of groups on child-directed play	Number of groups—child-directed play	Mappings (Ortega et al., 2009)	Ortega et al. (2009)
	Group size on child-directed play	Size of group—child-directed play	Mappings (Ortega et al., 2009)	Ortega et al. (2009)
	Group size on teacher-directed play	Size of group—teacher-directed play	Mappings (Ortega et al., 2009)	Ortega et al. (2009)
	Group composition on teacher-directed play	Group composition—teacher-directed play	Mappings (Ortega et al., 2009)	Ortega et al. (2009)

#### Social-Emotional Outcomes

Improvements were found for social-emotional competence (Chinekesh et al., 2014; Flook et al., 2015; Cheng and Ray, 2016), specifically on self-awareness (Chinekesh et al., 2014), empathy (Chinekesh et al., 2014; Cheng and Ray, 2016), emotion recognition (Richard et al., 2019), and dysfunctional emotion regulation strategies (Richard et al., 2019). Improvements were also found for social interaction (Hashemi et al., 2012; Goldstein and Lerner, 2017; Tersi and Matsouka, 2020), social cooperation (Hashemi et al., 2012; Loukatari et al., 2019; Tersi and Matsouka, 2020), social independence (Hashemi et al., 2012; Tersi and Matsouka, 2020), social assertion (Loukatari et al., 2019), friendship skills (Ozyurek et al., 2015), sharing (Flook et al., 2015), and peer relations problems (Deneault et al., 2020). No differences were found in Theory of Mind (Goldstein and Lerner, 2017), emotion expression (Lee et al., 2020), emotion identification (Richard et al., 2019), functional emotion regulation strategies (Richard et al., 2019), delay of gratification (Flook et al., 2015; Robinson et al., 2016; Murray et al., 2018), cognitive flexibility (Flook et al., 2015), altruism (Goldstein and Lerner, 2017; Richard et al., 2019), peer acceptance (Anna et al., 2016), and social avoidance (Richard et al., 2019). Contradictory results were found for emotion attribution (Goldstein and Lerner, 2017; Richard et al., 2019), emotion regulation (Flook et al., 2015; Ozyurek et al., 2015; Goldstein and Lerner, 2017; Richard et al., 2019), self-regulation (Chinekesh et al., 2014; Cheng and Ray, 2016; Duman and Ozkur, 2019; Loukatari et al., 2019), inhibitory control (Flook et al., 2015; Murray et al., 2018; Solomon et al., 2018), social competence (Lobo and Winsler, 2006; Hashemi et al., 2012; Chinekesh et al., 2014; Flook et al., 2015; Biber, 2016; Cheng and Ray, 2016; Solomon et al., 2018; Loukatari et al., 2019; Tersi and Matsouka, 2020), and prosocial behavior (Flook et al., 2015; Goldstein and Lerner, 2017; Richard et al., 2019; Deneault et al., 2020).

#### Child's Play

Improvements were found regarding play (Ortega et al., 2009), specifically on interaction with communication on child-directed play (Ortega et al., 2009), number of groups on teacher-directed play (Ortega et al., 2009), interaction with communication on teacher-directed play (Ortega et al., 2009), play disruption (Lee et al., 2020), play disconnection (Lee et al., 2020), play interaction (Lee et al., 2020), happiness after play (Lee et al., 2020), play extent (Lee et al., 2020), play intensity (Lee et al., 2020), and play skill (Lee et al., 2020). No differences were found in the group composition on child-directed play (Ortega et al., 2009), number of groups on child-directed play (Ortega et al., 2009), group size on child-directed play (Ortega et al., 2009), group size on teacher-directed play (Ortega et al., 2009), and group composition on teacher-directed play (Ortega et al., 2009).

#### Child's Behaviors

Improvements were reported in general internalizing behaviors (Lobo and Winsler, 2006; Loukatari et al., 2019; Deneault et al., 2020; Tersi and Matsouka, 2020), and general externalizing behaviors (Lobo and Winsler, 2006; Loukatari et al., 2019; Tersi and Matsouka, 2020), specifically on behavior problems (Deneault et al., 2020; Tersi and Matsouka, 2020), and hyperactivity (Loukatari et al., 2019; Deneault et al., 2020). No differences were found on anxiety/withdrawal (Solomon et al., 2018) and anger/aggression (Solomon et al., 2018; Richard et al., 2019).

### Methodological Quality of Selected Studies

Detailed information on methodological quality is presented on Table 3. Of the overall study assessments conducted with the PEDro scale (*n* = 19), which scores ranged from 3 to 7 (mean score, 4.5), 53% of studies (*n* = 10) were rated with a low quality of rigor (scored <5) (Ortega et al., 2009; Hashemi et al., 2012; Chinekesh et al., 2014; Flook et al., 2015; Ozyurek et al., 2015; Biber, 2016; Duman and Ozkur, 2019; Loukatari et al., 2019; Deneault et al., 2020; Tersi and Matsouka, 2020), and the rest (47%, *n* = 9) were rated with a high quality of rigor (scored 5 or higher) (Anna et al., 2016; Cheng and Ray, 2016; Robinson et al., 2016; Goldstein and Lerner, 2017; Murray et al., 2018; Solomon et al., 2018; Richard et al., 2019; Lee et al., 2020). All the studies met the criteria “groups similar at baseline” and “point measures and variability.” More than half of the studies satisfied the criteria “random allocation,” “follow-up,” and “between-group comparisons.” Only 3 studies met the criterion “blind assessor.” Two studies satisfied the criteria “blind therapist” and “intention-to-treat analysis,” and 1 study fulfilled the criteria “concealed allocation.” The criterion “blind subject” was not satisfied in any study.

**Table 3:** Methodological quality of the studies.

**Study**	**Eligibility criteria**	**Random allocation**	**Concealed allocation**	**Groups similar at baseline**	**Blind subject**	**Blind therapist**	**Blind assessor**	**Follow-up**	**Intention-to-treat analysis**	**Between-group comparisons**	**Point measures and variability**	**PEDro score**
Lobo and Winsler (2006)	1	1	0	1	0	0	1	1	0	1	1	6
Ortega et al. (2009)	0	0	0	1	0	0	0	0	0	1	1	3
Hashemi et al. (2012)	0	1	0	1	0	0	0	0	0	1	1	4
Chinekesh et al. (2014)	0	1	0	1	0	0	0	0	0	0	1	3
Flook et al. (2015)	0	1	0	1	0	0	0	0	0	1	1	4
Ozyurek et al. (2015)	0	0	0	1	0	0	0	1	0	1	1	4
Anna et al. (2016)	0	1	0	1	0	0	0	1	0	1	1	5
Biber (2016)	0	0	0	1	0	0	0	1	0	1	1	4
Cheng and Ray (2016)	1	1	0	1	0	0	0	1	0	1	1	5
Robinson et al. (2016)	0	1	0	1	0	0	0	1	0	1	1	5
Goldstein and Lerner (2017)	0	1	0	1	0	1	1	1	0	1	1	7
Murray et al. (2018)	1	1	0	1	0	1	0	1	0	1	1	6
Solomon et al. (2018)	1	1	0	1	0	0	1	0	1	1	1	6
Duman and Ozkur (2019)	0	0	0	1	0	0	0	1	0	1	1	4
Loukatari et al. (2019)	0	1	0	1	0	0	0	0	0	0	1	3
Richard et al. (2019)	0	1	0	1	0	0	0	1	0	1	1	5
Deneault et al. (2020)	0	0	0	1	0	0	0	1	0	0	1	3
Lee et al. (2020)	0	0	1	1	0	0	0	0	1	1	1	5
Tersi and Matsouka (2020)	0	0	0	1	0	0	0	0	0	1	1	3
Total	4	12	1	19	0	2	3	11	2	16	19	

### Level of the Scientific Evidence

**Figure 2:**
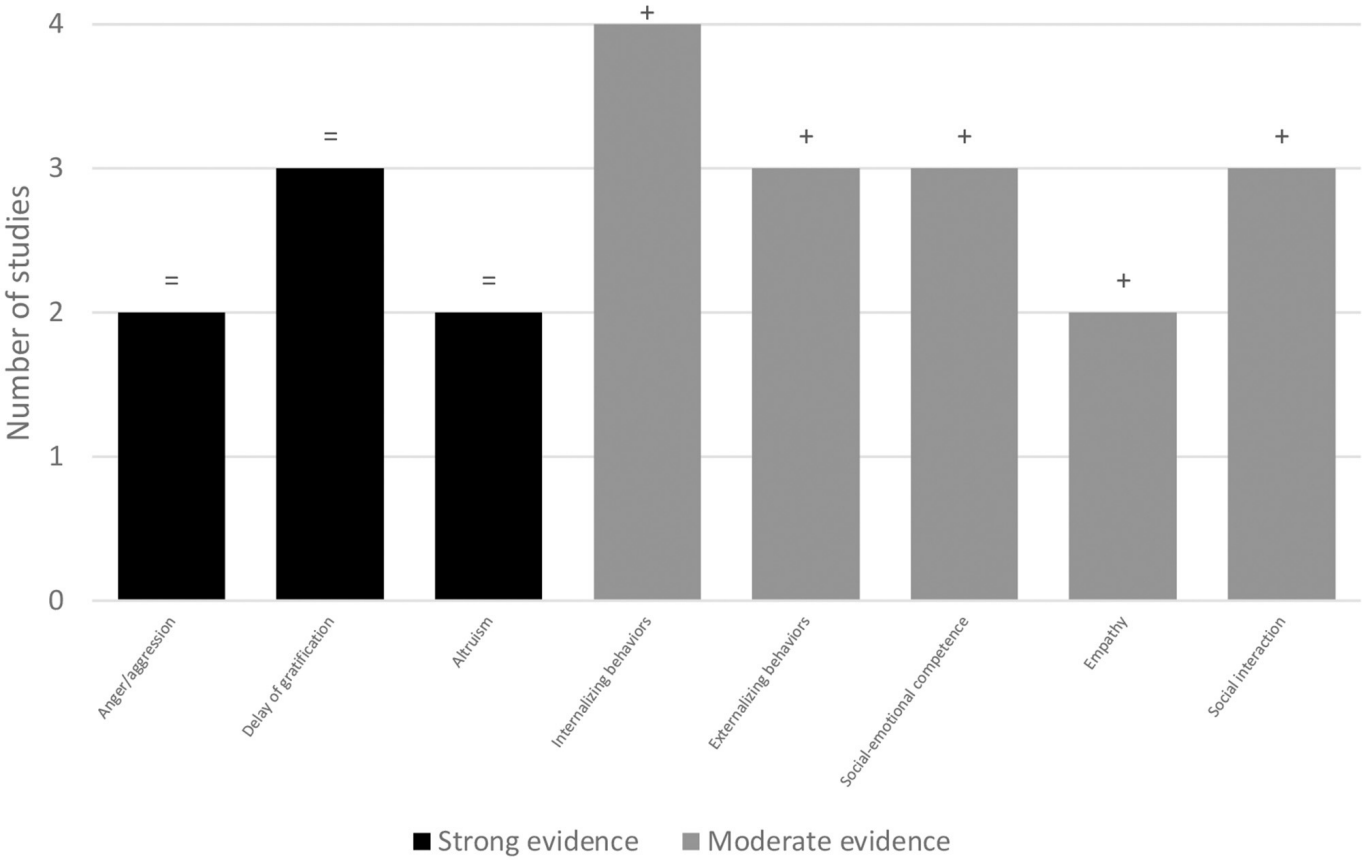
Key outcomes in strong and moderate level of strength evidence. +, positive results; =, no differences.

Figure 2 shows the strong and moderate level of the scientific evidence of key outcomes in BOI.

Regarding social-emotional outcomes, strong evidence was found for the lack of effects: in delay of gratification comparing with inactive (Flook et al., 2015; Robinson et al., 2016) and both inactive and active groups (Murray et al., 2018), provided by 1 low-quality RCT and 2 high-quality RCTs; and altruism comparing with two active (Goldstein and Lerner, 2017) and 1 inactive (Richard et al., 2019) groups, provided by 2 high-quality RCTs. Moderate evidence was found for improving: social-emotional competence comparing with inactive groups (Chinekesh et al., 2014; Flook et al., 2015; Cheng and Ray, 2016), provided by 2 low-quality RCTs and 1 high-quality RCT; empathy comparing with inactive groups (Chinekesh et al., 2014; Cheng and Ray, 2016), provided by 1 low-quality RCT and 1 high-quality RCT; and social interaction comparing with inactive (Hashemi et al., 2012; Tersi and Matsouka, 2020) and 2 active groups (Goldstein and Lerner, 2017), provided by 2 low-quality RCTs and 1 high-quality RCT. Limited evidence was found for improving emotion recognition and dysfunctional emotion regulation strategies comparing with an inactive group (Richard et al., 2019), provided by 1 high-quality RCT; for improving social cooperation comparing with inactive groups (Hashemi et al., 2012; Loukatari et al., 2019; Tersi and Matsouka, 2020), provided by 3 low-quality RCTs; and social independence comparing with inactive groups (Hashemi et al., 2012; Tersi and Matsouka, 2020), provided by 2 low-quality RCTs. Limited evidence was also found for the lack of effects in: Theory of Mind comparing with 2 active groups (Goldstein and Lerner, 2017), provided by 1 high-quality RCT; emotion expression comparing with an inactive group (Lee et al., 2020), provided by 1 high-quality RCT; emotion identification and functional emotion regulation strategies comparing with an inactive group (Richard et al., 2019), provided by 1 high-quality RCT; peer acceptance comparing with an inactive group (Anna et al., 2016), provided by 1 high-quality RCT; and social avoidance comparing with an inactive group (Richard et al., 2019), provided by 1 high-quality RCT. No evidence was found for the rest of the outcomes. Detailed information is presented in Table 4.

**Table 4:** Level of the scientific evidence of the effects of BOI on social-emotional competence.

**Outcomes**	**Study**	Results*	**PEDro scale**	**Level of evidence**
**Category: Social-emotional outcomes**				
Social-emotional competence	Chinekesh et al. (2014)	Positive	3	Moderate evidence
	Flook et al. (2015)	Positive	4	
	Cheng and Ray (2016)	Positive	5	
Self-awareness	Chinekesh et al. (2014)	Positive	3	No evidence
Empathy	Chinekesh et al. (2014)	Positive	3	Moderate evidence
	Cheng and Ray (2016)	Positive	5	
Theory of Mind	Goldstein and Lerner (2017)	Negative	7	Limited evidence
Emotion expression	Lee et al. (2020)	Negative	5	Limited evidence
Emotion identification	Richard et al. (2019)	Negative	5	Limited evidence
Emotion recognition	Richard et al. (2019)	Positive	5	Limited evidence
Emotion attribution	Goldstein and Lerner (2017)	Negative	7	No evidence
	Richard et al. (2019)	Positive	5	
	Richard et al. (2019)	Positive	5	
	Richard et al. (2019)	Negative	5	
Emotion regulation	Flook et al. (2015)	Positive	4	No evidence
	Ozyurek et al. (2015)	Positive	4	
	Goldstein and Lerner (2017)	Positive	7	
	Richard et al. (2019)	Negative	5	
Functional emotion regulation strategies	Richard et al. (2019)	Negative	5	Limited evidence
Dysfunctional emotion regulation strategies	Richard et al. (2019)	Positive	5	Limited evidence
Self-regulation	Chinekesh et al. (2014)	Positive	3	No evidence
	Cheng and Ray (2016)	Negative	5	
	Duman and Ozkur (2019)	Positive	4	
	Loukatari et al. (2019)	Positive	3	
Delay of gratification	Flook et al. (2015)	Negative	4	Strong evidence
	Robinson et al. (2016)	Negative	5	
	Murray et al. (2018)	Negative	6	
Inhibitory control	Flook et al. (2015)	Negative	4	No evidence
	Murray et al. (2018)	Negative	6	
	Solomon et al. (2018)	Positive	6	
	Solomon et al. (2018)	Negative	6	
Cognitive flexibility	Flook et al. (2015)	Negative	4	No evidence
Social competence	Lobo and Winsler (2006)	Positive	6	No evidence
	Hashemi et al. (2012)	Positive	4	
	Chinekesh et al. (2014)	Positive	3	
	Chinekesh et al. (2014)	Positive	3	
	Flook et al. (2015)	Positive	4	
	Biber (2016)	Positive	4	
	Cheng and Ray (2016)	Positive	5	
	Solomon et al. (2018)	Negative	6	
	Loukatari et al. (2019)	Positive	3	
	Tersi and Matsouka (2020)	Positive	3	
Social interaction	Hashemi et al. (2012)	Positive	4	Moderate evidence
	Goldstein and Lerner (2017)	Positive	7	
	Tersi and Matsouka (2020)	Positive	3	
Social cooperation	Hashemi et al. (2012)	Positive	4	Limited evidence
	Loukatari et al. (2019)	Positive	3	
	Tersi and Matsouka (2020)	Positive	3	
Social independence	Hashemi et al. (2012)	Positive	4	Limited evidence
	Tersi and Matsouka (2020)	Positive	3	
Social assertion	Loukatari et al. (2019)	Positive	3	No evidence
Friendship skills	Ozyurek et al. (2015)	Positive	4	No evidence
Altruism	Goldstein and Lerner (2017)	Negative	7	Strong evidence
	Richard et al. (2019)	Negative	5	
Sharing	Flook et al. (2015)	Positive	4	No evidence
Peer acceptance	Anna et al. (2016)	Negative	5	Limited evidence
Social avoidance	Richard et al. (2019)	Negative	5	Limited evidence
Peer relations problems	Deneault et al. (2020)	Positive	3	No evidence
Prosocial behavior	Flook et al. (2015)	Positive	4	No evidence
	Goldstein and Lerner (2017)	Negative	7	
	Goldstein and Lerner (2017)	Negative	7	
	Richard et al. (2019)	Negative	5	
	Deneault et al. (2020)	Positive	3	
**Category: Child's play**				
Play	Ortega et al. (2009)	Positive	3	No evidence
Interaction with communication on child-directed play	Ortega et al. (2009)	Positive	3	No evidence
Number of groups on teacher-directed play	Ortega et al. (2009)	Positive	3	No evidence
Interaction with communication on teacher-directed play	Ortega et al. (2009)	Positive	3	No evidence
Play disruption	Lee et al. (2020)	Positive	5	Limited evidence
Play disconnection	Lee et al. (2020)	Positive	5	Limited evidence
Play interaction	Lee et al. (2020)	Positive	5	Limited evidence
Happiness after play	Lee et al. (2020)	Positive	5	Limited evidence
Play extent	Lee et al. (2020)	Positive	5	Limited evidence
Play intensity	Lee et al. (2020)	Positive	5	Limited evidence
Play skill	Lee et al. (2020)	Positive	5	Limited evidence
Group composition on child-directed play	Ortega et al. (2009)	Negative	3	No evidence
Number of groups on child-directed play	Ortega et al. (2009)	Negative	3	No evidence
Group size on child-directed play	Ortega et al. (2009)	Negative	3	No evidence
Group size on teacher-directed play	Ortega et al. (2009)	Negative	3	No evidence
Group composition on teacher-directed play	Ortega et al. (2009)	Negative	3	No evidence
**Category: Child's Behaviors**				
General internalizing behaviors	Lobo and Winsler (2006)	Positive	6	Moderate evidence
	Loukatari et al. (2019)	Positive	3	
	Deneault et al. (2020)	Positive	3	
	Tersi and Matsouka (2020)	Positive	3	
Anxiety/withdrawal	Solomon et al. (2018)	Negative	6	Limited evidence
General externalizing behaviors	Lobo and Winsler (2006)	Positive	6	Moderate evidence
	Loukatari et al. (2019)	Positive	3	
	Tersi and Matsouka (2020)	Positive	3	
Anger/aggression	Solomon et al. (2018)	Negative	6	Strong evidence
	Richard et al. (2019)	Negative	5	
Behavior problems	Deneault et al. (2020)	Positive	3	Limited evidence
	Tersi and Matsouka (2020)	Positive	3	
Hyperactivity	Loukatari et al. (2019)	Positive	3	Limited evidence
	Deneault et al. (2020)	Positive	3	

*Positive results if the intervention program was more effective than the control condition with regard to outcomes measured; negative results if there were no statistically significant differences between the intervention group and the control condition with regard to outcomes measured or if the control condition was more effective*.

Concerning child's play, limited evidence was found for improving play disruption, play disconnection, play interaction, happiness after play, play extent, play intensity, and play skill, comparing with an inactive group (Lee et al., 2020), provided by 1 high-quality RCT. No evidence was found for improving play, interaction with communication on child-directed play, number of groups on teacher-directed play, and interaction with communication on teacher-directed play (Ortega et al., 2009). No evidence was also found for the lack of effects in group composition on child-directed play, number of groups on child-directed play, group size on child-directed and teacher-directed play, and group composition on teacher-directed play comparing with an inactive group (Ortega et al., 2009), provided by 1 low-quality RCT.

Regarding child's behavior's, strong evidence was found for the lack of effects in anger/aggression, comparing with an active control group (Solomon et al., 2018) or with an inactive group (Richard et al., 2019), provided by 2 high-quality RCTs. Moderate evidence was found for: decreased general internalizing behaviors comparing with inactive groups (Lobo and Winsler, 2006; Loukatari et al., 2019; Deneault et al., 2020; Tersi and Matsouka, 2020), provided by 1 high-quality RCT and 3 low-quality RCTs; and decreased general externalizing behaviors comparing with inactive groups (Lobo and Winsler, 2006; Loukatari et al., 2019; Tersi and Matsouka, 2020), provided by 1 high-quality RCT and 2 low-quality RCTs. Limited evidence was found for: decreased behavior problems comparing with inactive groups (Deneault et al., 2020; Tersi and Matsouka, 2020), provided by 2 low-quality RCTs; decreased hyperactivity comparing with inactive groups (Loukatari et al., 2019; Deneault et al., 2020), provided by 2 low-quality RCTs; and for the lack of effects in anxiety/withdrawal comparing with an active group (Solomon et al., 2018), provided by 1 high-quality RCT.

## Discussion

To date, this is the first systematic review to locate and synthesize the effects of BOI implemented in educational contexts on preschoolers' social-emotional competence. Over the past 14 years, research in this area has been growing, evidencing the relevance of implementing BOI in preschool education contexts regarding social-emotional competence. Although the first RCT was published in 2006, most RCTs were carried out between 2016 and 2020, showing that research on the implementation of this type of intervention in the educational context is relatively recent.

This systematic review analyzed different BOI programs with different lengths, frequencies, and durations of sessions. Despite the difficulty to identify the ideal intervention dosage, the emerging consensus among researchers is that children who received more dosage exhibited greater increases in treatment outcomes (Lochman et al., 2006; Zhai et al., 2010; Yazejian et al., 2015). However, the findings of this systematic review do not support these conclusions. For example, in the study conducted by Lee et al. (2020) the BOI program lasted for 1 week, 5 days/week with a duration of 70 mins each session, and significant effects were observed in most of the outcomes (e.g., happiness after play, play disruption, play disconnection). Otherwise, in the study conducted by Solomon et al. (2018), the 60-week BOI program integrated into the preschool curriculum showed positive effects only in one of the analyzed outcomes. Nevertheless, we should consider that the participants' age and the outcomes analyzed in each study may have influenced these findings.

Regarding the social-emotional outcomes, one should note that, so far, many of the competences previously investigated with older children (Denham, 2006; Collaborative for Academic Social and Emotional Learning, 2013) have not been studied regarding preschool-aged children, including problem-solving skills (Ramani and Brownell, 2014), self-efficacy (Bistagani and Najafi, 2017), and self-confidence (Liu et al., 2015).

Some of the assessment instruments used in the included studies were parent and teacher reported. The use of parent reports is based on the idea that parents see and know children in various contexts and situations; therefore, they can observe their children's behaviors across multiple situations (Crozier and Badawood, 2009). However, parents may be biased when reporting their children's behaviors (e.g., they may be motivated to positively portray their children's behavior) (Gartstein et al., 2012). Regarding teachers' reported measures, this might not have such bias because teachers can observe children's behaviors in the school context and compare a particular child's behavior with children of the same age (Crozier and Badawood, 2009). Otherwise, teachers' perspectives on children's behaviors were foremost based on their interactions in the classroom, which means that some relevant behaviors that occur in other contexts (e.g., recess time) may go unnoticed.

On average, the methodological quality of studies was low, which goes against what was expected since most studies are recent (after the year 2016). One of the items less satisfied was criterion related to blinding, where the person (assessor, therapist, and/or subjects) in question must not know which group the subject had been allocated (de Morton, 2009). In experimental studies, where the therapists must implement a particular intervention program, it is difficult to achieve this criterion, often because blinding the therapist and subject to treatment is non-feasible (Park, 2013; Armijo-Olivo et al., 2017; Renjith, 2017). In the two studies that reported blinding of the therapist, the same therapist implemented different intervention programs, and he/she was blind to the study's hypothesis (Goldstein and Lerner, 2017; Murray et al., 2018). Since BOI integrates different therapeutic approaches such as exercise and physical activity, the blinding of subjects is difficult (Wahbeh et al., 2008; Renjith, 2017; Hecksteden et al., 2018). Despite this, this criterion was omitted by the authors of the studies and therefore was not satisfied. Regarding assessors blinding, only three studies explicitly state that the assessors were blind to the condition of the assessed child. This criterion was not satisfied in the remaining studies, probably because measures were teacher- and/or parent-reported.

Regarding social-emotional outcomes, there was strong evidence that BOI have no effects in delay of gratification (Flook et al., 2015; Robinson et al., 2016; Murray et al., 2018), and altruism (Goldstein and Lerner, 2017; Richard et al., 2019). The literature suggests that delay of gratification is related to altruism (Osiński et al., 2017; Gruen et al., 2020; Koomen et al., 2020), demonstrating that altruistic children are capable of delaying gratification for a cooperative goal (Koomen et al., 2020). Since children under 5 years old demonstrate a marked lack of this ability, and only throughout the fifth year they exhibit cognitive strategies needed for delaying gratification (Twito et al., 2019), in Flook et al.'s (2015) and Robinson et al.'s (2016) studies the average age of the children was 4 years old, which explains the lack of positive effects. Otherwise, in Murray et al. (2018) study the mean age of the participants was 6.24, but the lack of positive effects may be due to the short intervention duration and frequency (1 week, 3 × 11′ per week).

There was moderate evidence that BOI improve social-emotional competence (Chinekesh et al., 2014; Flook et al., 2015; Cheng and Ray, 2016), such as empathy (Chinekesh et al., 2014; Cheng and Ray, 2016), and social interaction (Hashemi et al., 2012; Goldstein and Lerner, 2017; Tersi and Matsouka, 2020). These results were expected since more empathic children exhibit better social behaviors (Findlay et al., 2006; Paulus and Leitherer, 2017; Hirn et al., 2019).

There was limited evidence for the positive effects of BOI in emotion recognition (Richard et al., 2019), dysfunctional emotion regulation strategies (Richard et al., 2019), social cooperation (Hashemi et al., 2012; Loukatari et al., 2019; Tersi and Matsouka, 2020), and social independence (Hashemi et al., 2012; Tersi and Matsouka, 2020). Possibly, the significant body and emotional experiences provided by BOI (Probst et al., 2010; Mehling et al., 2011; Robins et al., 2012), facilitate the development of emotion recognition and regulation. These abilities are essential to social interactions (Oerlemans et al., 2014), and are predictors of cooperative social behaviors (Denham et al., 2003; Cole et al., 2009). Besides, there was limited evidence for the lack of effects in Theory of Mind (Goldstein and Lerner, 2017), emotion expression (Lee et al., 2020), emotion identification (Richard et al., 2019), functional emotion regulation strategies (Richard et al., 2019), peer acceptance (Anna et al., 2016), and social avoidance (Richard et al., 2019). The nonexistence of positive effects may be due to the intervention programs characteristics (e.g., type of BOI, and duration or frequency of intervention program), the age of participants, or the assessment instruments used in each study (as teacher- or parent-reported measures may lead to different outcomes). There was no evidence for BOI to improve self-awareness (Chinekesh et al., 2014), and for the lack of effects regarding cognitive flexibility (Flook et al., 2015).

No evidence was found for outcomes with contradictory results, such as emotion attribution, emotion regulation, self-regulation, inhibitory control, social competence, and prosocial behavior, which calls into question the effectiveness of BOI in some important social-emotional outcomes. Regarding emotion attribution, improvements were found by Richard et al. (2019) in two different assessments and no differences in other assessment. In the study conducted by Goldstein and Lerner (2017), a decreased in this outcome was observed. Richard et al. (2019) implemented an 11-week intervention program with 1 session of 60 mins per week, compared with an inactive group. In contrast, Goldstein and Lerner (2017) implemented an 8-week intervention program with 3 sessions of 30 mins per week, comparing with 2 active groups. Therefore, these inconsistencies in outcomes may be due to the different characteristics of the intervention programs and/or comparison groups.

The study of Richard et al. (2019) showed no differences in emotion regulation. Otherwise, Flook et al. (2015), Ozyurek et al. (2015), Goldstein and Lerner (2017), and colleagues found improvements in this outcome after the intervention programs. These contradictory results may be due to the sample sizes of the studies since in the study where no differences were observed, the sample size was the smallest (*n* = 19 vs. *n* > 42).

In the study conducted by Cheng and Ray (2016), no differences were found in self-regulation after 8 weeks of BOI program. In contrast, the studies of Chinekesh et al. (2014), Duman and Ozkur (2019), and Loukatari et al. (2019), found improvements in self-regulation after 5, 12 and 4 weeks, respectively, of BOI programs. Cheng and Ray (2016) study involved two 30-mins sessions per week, whereas the other studies involved three 90-mins sessions, five 45–60-min sessions, and three 30-mins sessions per week, respectively. These differences in the frequency and duration of the sessions suggest that the dosage of intervention is critical for self-regulation improvements.

Solomon et al. (2018) found improvements in inhibitory control in one of the 2 different assessments used to evaluate this outcome. In turn, Flook et al. (2015) and Murray et al. (2018) also measured inhibitory control but found no differences. These contradictory results may be related to sample sizes and/or the age of the participants' differences. In the studies conducted by Flook et al. (2015), Murray et al. (2018), and colleagues, the sample sizes were 68 and 101, and the mean age of the participants was 4.67 and 6.24, respectively, whereas in the study conducted by Solomon et al. (2018) participated 256 children with 3–4 years of age, which represents the largest sample among these studies, as well as with younger participants. Moreover, the contradictory findings for the same outcome found by Solomon et al. (2018) may be explained by the different assessment instruments. The improvements on inhibitory control detected by an instrument (Ponitz et al., 2009) that involved more physical activity and fewer test trials suggests that this instrument may be more optimally suited to tap early, subtle improvements in inhibitory control. These characteristics may be noteworthy to consider when choosing an assessment instrument for preschoolers.

Improvements were found for social competence in eight of the nine studies that assessed this outcome (Lobo and Winsler, 2006; Hashemi et al., 2012; Chinekesh et al., 2014; Flook et al., 2015; Biber, 2016; Cheng and Ray, 2016; Loukatari et al., 2019; Tersi and Matsouka, 2020). In the study conducted by Solomon et al. (2018), where no differences were found in social competence, the lack of positive changes may be due to the similarity of the experimental condition and active control group. However, according to Karlsson and Bergmark (2014), this cannot be interpreted as a lack of treatment efficacy.

Finally, contradictory results were also found for prosocial behavior. While improvements were observed by Flook et al. (2015) and Deneault et al. (2020), no improvements were found by Goldstein and Lerner (2017) and Richard et al. (2019). Such differences may be due to the different assessment instruments used. That is, in Flook et al.'s (2015) and Deneault et al.'s (2020) studies, the instruments were reported by teachers, whereas in Goldstein and Lerner (2017) and Richard et al. (2019), the measures were reported by the researchers or applied to children, respectively.

Regarding child's play, there was limited evidence that BOI improves play disruption, play disconnection, play interaction, happiness after play, play extent, play intensity, and play skill (Lee et al., 2020). There was no evidence for the improvements in play, interaction with communication in child-directed play, number of groups and interaction with communication in teacher-directed play (Ortega et al., 2009). Likewise, there was no evidence for the lack of effects in group composition, number of groups and group size in child-directed play, and in group size and group composition in teacher-directed play (Ortega et al., 2009). This lack of scientific evidence of these outcomes is related to the low quality of the RCT that provided these outcomes, supporting the need for new studies to clearly understand the scientific evidence of the effects of BOI on children's play.

Regarding child's behaviors, there was strong evidence that BOI have no effects in anger/aggression (Solomon et al., 2018; Richard et al., 2019). Instead, we found moderate evidence for the decrease in general externalizing behaviors (Lobo and Winsler, 2006; Loukatari et al., 2019; Tersi and Matsouka, 2020). As we observed positive results in general externalizing behaviors, a decrease in anger/aggression was expected (Achenbach et al., 2016; Willner et al., 2016). Contrary to these results, there was limited evidence for the decrease of behavior problems (Deneault et al., 2020; Tersi and Matsouka, 2020), and hyperactivity (Loukatari et al., 2019; Deneault et al., 2020), as expected since these variables fit into the general externalizing behaviors.

There was moderate evidence for the positive effects of BOI in general internalizing behaviors (Lobo and Winsler, 2006; Loukatari et al., 2019; Deneault et al., 2020; Tersi and Matsouka, 2020). However, when specific internalizing behaviors were examined, it was found limited evidence for the lack of effects of BOI in anxiety/withdrawal (Solomon et al., 2018). This lack of positive changes may be due to the similarity of the experimental condition and active control group, which cannot be interpreted as a lack of treatment efficacy (Karlsson and Bergmark, 2014).

## Study Limitations

There are several limitations in this systematic review: the inclusion criteria requiring a BOI implemented in the school context limited the number of selected studies. We did not determine the validity and reliability of the instruments, the qualifications of BOI therapists, or the appropriateness of statistical analyses. The methodological quality assessment can also be considered a limitation since the items are only satisfied when the study clearly states whether the criterion is satisfied or not. Levels of evidence measured by BES may also be a limitation. There may be studies of high methodological quality showing positive effects in the analyzed outcome, but the existence of 1 low-quality study with contradictory results take to no evidence of the effects in this outcome.

## Conclusions

The part I of this systematic review showed that there was strong evidence that BOI do not improve delay of gratification, altruism, and anger/aggression. Besides, there was moderate evidence that BOI improve social-emotional competence, empathy, social interaction, internalizing and externalizing behaviors. There was limited evidence that BOI improve emotion recognition, dysfunctional emotion regulation strategies, social cooperation, social independence, play disruption, play disconnection, play interaction, happiness after play, play extent, play intensity, play skill, behavior problems, and hyperactivity. There was also limited evidence that BOI do not improve Theory of Mind, emotion expression, emotion identification, functional emotion regulation strategies, peer acceptance, social avoidance, and anxiety/withdrawal. There was no evidence of the effectiveness of BOI in the remaining social-emotional outcomes.

The present systematic review supports the need for further experimental studies that evaluate the effectiveness of BOI in preschoolers' social-emotional competence, with a greater methodological quality (e.g., blinding of subjects, therapists, and assessors). Future investigations must not omit important data such as eligibility criteria, whether the allocation was random, whether the subjects, therapists, and assessors were blind to the study hypotheses, or whether there was an intention-to-treat. Further research must establish the optimal frequency, duration, and intensity of BOI programs to lead to effective interventions. Overall, the first part of this systematic review showed that BOI might be an effective intervention to improve specific social-emotional competences in preschool children. However, knowing the effectiveness of which type of BOI on preschoolers' social-emotional competence is also of paramount importance. That is the aim of the second part of this systematic review.

## Data Availability Statement

The original contributions presented in the study are included in the article/supplementary material, further inquiries can be directed to the corresponding authors.

## Author Contributions

AD, JM, and GV contributed to the conception and design of the study. AD and GV selected the studies and extracted the data. AD and AC-F assessed independently the methodological quality and the level of scientific evidence of the studies. The third reviewer (JM) was consulted to resolve disagreements. AD wrote the first draft of the manuscript. GV supervised the study. All authors contributed to manuscript revision, read, and approved the submitted version.

## Funding

This work is funded by national funds through the Foundation for Science and Technology, under the project UIDP/04923/2020.

## Conflict of Interest

The authors declare that the research was conducted in the absence of any commercial or financial relationships that could be construed as a potential conflict of interest.

## Publisher's Note

All claims expressed in this article are solely those of the authors and do not necessarily represent those of their affiliated organizations, or those of the publisher, the editors and the reviewers. Any product that may be evaluated in this article, or claim that may be made by its manufacturer, is not guaranteed or endorsed by the publisher.
